# Enhancing Immunomodulatory Function of Red Ginseng Through Fermentation Using *Bifidobacterium animalis* Subsp. *lactis* LT 19-2

**DOI:** 10.3390/nu11071481

**Published:** 2019-06-28

**Authors:** Jae Hwan Kim, Eun-Hee Doo, Minju Jeong, Seungil Kim, Yun-Yeol Lee, Jaesik Yang, Ji Su Lee, Jong Hun Kim, Ki Won Lee, Chul Sung Huh, Sanguine Byun

**Affiliations:** 1 Department of Agricultural Biotechnology, Seoul National University, Seoul 08826, Korea; 2 Research Institute of Eco-friendly Livestock Science, Institute of Green-Bio Science and Technology, Seoul National University, Pyeongchang 25354, Korea; 3 Graduate School of International Agricultural Technology, Seoul National University, Pyeongchang 25354, Korea; 4 Food Analysis Center, Korea Food Research Institute, Wanju 55365, Korea; 5 Division of Bioengineering, Incheon National University, Incheon 22012, Korea; 6 Department of Food Science and Biotechnology, Sungshin University, Seoul 01133, Korea; 7 Research Institute of Agriculture and Life Sciences, Seoul National University, Seoul 08826, Korea

**Keywords:** immunomodulation, red ginseng, fermentation, ginsenosides

## Abstract

Removal of sugar moieties from ginsenosides has been proposed to increase their biological effects in various disease models. In order to identify strains that can increase aglycone contents, we performed a screening using bacteria isolated from the feces of infants focusing on acid tolerance and β-glucosidase activity. We isolated 565 bacteria and selected *Bifidobacterium animalis* subsp. *lactis* LT 19-2 (LT 19-2), which exhibited the highest β-glucosidase activity with strong acid tolerance. As red ginseng (RG) has been known to exert immunomodulatory functions, we fermented RG using LT 19-2 (FRG) and investigated whether this could alter the aglycone profile of ginsenosides and improve its immunomodulatory effect. FRG increased macrophage activity more potently compared to RG, demonstrated by higher TNF-α and IL-6 production. More importantly, the FRG treatment stimulated the proliferation of mouse splenocytes and increased TNF-α levels in bone marrow-derived macrophages, confirming that the enhanced immunomodulatory function can be recapitulated in primary immune cells. Examination of the molecular mechanism revealed that F-RG could induce phosphorylations of ERK, p38, JNK, and NF-κB. Analysis of the ginsenoside composition showed a decrease in Rb1, Re, Rc, and Rb3, accompanied by an increase in Rd, Rh1, F2, and Rg3, the corresponding aglycone metabolites, in FRG compared to RG. Collectively, LT 19-2 maybe used as a probiotic strain to improve the bioactivity of functional foods through modifying the aglycone/glycoside profile.

## 1. Introduction

Red ginseng (RG) is a functional food/herbal medicine generated through processing ginseng (*Panax ginseng*) by steaming. Specifically, RG is manufactured by conducting multiple cycles of steaming raw ginseng at 95–100 °C [1,2] for at least 80–100 min, followed by drying at 45–55 °C until the moisture content is lower than 15.5% [3]. Studies have reported the biological effects of RG in metabolic disease [4,5], skin aging [6,7], cancer [8,9], and immunological diseases [10,11].

RG has been reported to have superior bioactivity compared to white ginseng, which has been mainly attributed to the increase in the amount of ginsenosides with their sugar moieties removed [12,13]. The ginsenoside composition of steamed ginseng has been investigated in a number of studies. Multiple studies on steamed ginseng report that the steaming process produces various ginsenoside metabolites, which may function as major active compounds in RG [14,15,16,17,18,19]. For example, while analysis of ginsenoside contents in autoclaved American ginseng failed to detect Re, Rb1, Rc, Rb2, or Rd, their metabolites Rg3, Rk1, and Rg5 were detected [20]. A study comparing the ginsenoside profile found that white ginseng contained higher amounts of Rb1, Rc, Rb2, Rd, and Rg, whereas heat-processed ginseng contained higher amounts of their metabolites, ginsenosides Rg3, Rk1, and Rg5 [21]. More importantly, processed ginseng as well as specific metabolites only found in RG has been reported to display enhanced bioactivity. RG possessed stronger protective effects against diabetic renal damage in rats compared to white ginseng [20]. Compound K which is a well-known metabolite of Rb1 has shown superior anticancer effects compared to its parental ginsenosides [22]. Heat-processed ginseng, but not white ginseng, also stimulated innate immune function via upregulation of nuclear factor-kappa B (NF-κB) transcriptional activity, cytokine production, and the expression of major histocompatibility complex (MHC) class I and II molecules in RAW264.7 cells [21]. These studies demonstrate that formation of metabolites during RG processing can improve the health-beneficial effect of ginseng.

It has been suggested that transforming the chemical composition of RG using steaming processes or enzymatic conversions via fermentation, can cause an increase in the bioactivity [23,24,25]. Similarly, detaching sugar residues from ginsenosides has been shown to enhance their bioactivity [23,24,25]. Thus, many attempts have been conducted to transform ginsenosides into their aglycone forms [26,27,28].

To improve the pharmacological activity of functional resources, various organisms have been used for hydrolyzation. For hydrolyzing the sugar moieties of active compounds, the enzymatic activity of organisms is an important factor [29]. β-glucosidase, endoglucanase, and cellobiohydrolase are members of the cellulase enzyme system. β-glucosidase is known as a major enzyme that can promote the conversion of glycosides to aglycones. β-glucosidase catalyzes the hydrolysis of β-glucosidic linkages of aryl and alkyl β-glucosides, β-linked oligosaccharides, and other oligosaccharides, releasing sugars. β-glucosidase is involved in the final step of cellulose saccharification, converting cellobiose into glucose [30]. Thus, organisms with high β-glucosidase activity can be beneficial in the industry to efficiently detach the sugar residues of ginsenosides.

In this study, we aimed to isolate β-glucosidase-producing bacteria from infants. *Bifidobacterium* and *Lactobacillus* species are well-known health-promoting probiotics, which are common inhabitants of the infant intestinal tract. However, research shows that beneficial gut microbiota including *Bifidobacterium* and *Lactobacillus* species decline as humans age [31,32,33]. Previous studies also report the use of antibiotics as a reason for the decrease or removal of health promoting gut microbiota [34]. It has been reported that the diversity of gut microbiota of adults (over 20 years of age) is higher than that of children (1–4 years of age), while children have higher abundance of *Bifidobacterium* species than adults [35]. Thus, we screened bacteria from infant feces, which can be a relatively rich source for *Bifidobacterium* and *Lactobacillus* species with less complex bacterial community compared to adults. We focused on isolating *Bifidobacterium* or *Lactobacillus* species with high β-glucosidase activity and generated fermented RG using the selected bacteria (i.e., *Bifidobacterium animalis* subsp. *lactis* LT 19-2) and investigated its immune-regulatory function and mechanism.

## 2. Materials and Methods

### 2.1. Isolation and Identification of Probiotic Strains from Infant Feces

The experimental protocol was approved by the Seoul National University Institutional Review Board (IRB NO. 1605/003-006). Fecal samples were obtained from 30 Korean healthy infants aged less than 100 days. Briefly, 1 g of fresh feces was serially diluted 10-fold (to 10-9) with phosphate-buffered saline (PBS; pH 7.4; Mediatech Inc., Manassas, VA, USA), spread-plated onto de Man, Rogosa, and Sharpe (MRS) agar (Difco, Sparks, MD, USA) supplemented with 0.02% sodium azide (Sigma-Aldrich, St. Louis, MO, USA) and transoligosaccharide (TOS) propionate agar (Merck KGaA, Darmstadt, Germany), and incubated at 37 °C for 24–48 h under anaerobiosis (anaerobic chamber; Coy Laboratories, Ann Arbor, MI, USA). After incubation, single colonies showing phenotypic features similar to lactic acid bacteria (LAB) and bifidobacteria were subcultured anaerobically in MRS broth supplemented with 0.05% L-cysteine-HCl (Sigma-Aldrich) (MRSC) at 37 °C for 24 h to examine cell morphology and biochemical properties. Gram staining and catalase tests were carried out as described previously [36]. Gram-stained bacteria were observed using an ECLIPSE Ci-L microscope (Nikon, Tokyo, Japan). Pure cultures of single colonies were stored in 30% (v/v) glycerol solution at −80 °C. Culture media were sterilized by autoclaving at 121 °C for 15 min before use.

### 2.2. DNA Extraction and 16S Ribosomal RNA (16S rRNA) Gene Sequencing

The FastDNA SPIN Kit for Soil (MP Biomedicals, Solon, OH, USA) was used to extract DNA from the culture samples of selected strains. The quality and concentration of DNA were assessed using a SPECTROstar Nano instrument (BMG LABTECH GmbH, Ortenberg, Germany). To identify the strains, bacterial 16S rRNA gene sequencing was performed using the universal primers 27F (5′-AGA GTT TGA TCC TGG CTC AG-3′) and 1492R (5′-GGT TAC CTT GTT ACG ACT T-3′) (Macrogen Inc., Seoul, Korea) [36].

### 2.3. Acid Tolerance Tests

Acid tolerance of selected probiotic strains was tested via a modified method described previously [36]. To investigate the tolerance of selected strains to low pH, all inocula were cultivated anaerobically in MRSC at 37 °C for 24 h at 2 × 10^6^ colony-forming units (CFU)/mL, adjusted to pH 2.0 and 2.5 using 1 N HCl at 37 °C for 2 h. To test for cell viability, culture samples collected at 0 and 2 h were serially diluted in PBS, plated on MRSC agar plates, and incubated anaerobically at 37 °C for 48 h. The numbers of CFUs at 0 and 2 h were counted and used to calculate the bacterial survival rate (%) according to the following formula: Number of colonies at 2 h (CFU/mL)/number of colonies at 0 h (CFU/mL) × 100%.

### 2.4. Antibiotic Susceptibility Tests

Susceptibility of the bacteria strain to antibiotics was determined by the microdilution method described in M07-A9 of the National Committee for Clinical and Laboratory Standards Institute (NCCLSI) with some modification [37]. After anaerobic cultivation of strains in MRSC at 37 °C for 24 h, the cell pellets were washed twice with PBS and diluted until OD600 reached 0.3 on a McFarland standard of 1.0 in 2X LAB susceptibility test medium (LSM). Subsequently, the cell suspensions were diluted 300-fold with 2X LSM broth and 100 μL of the diluted cell suspensions were mixed with 100 μL 2X stock solutions of antibiotics, which were prepared from a two-fold dilution series. All reactions were performed in the same microplate. The stock solutions of ampicillin, kanamycin, tetracycline, erythromycin, gentamicin, and chloramphenicol stocks were prepared by serial dilution of 1 to 128 mg/L before use. After 48 h of anaerobic incubation at 37 °C, the minimal inhibitory concentrations (MICs) of antibiotics were determined. The MIC tests were conducted in triplicates. The microbiological MIC cut-off values were referenced from European Food Safety Authority (EFSA) guidelines for *Bifidobacterium* spp. Antibiotics used in this study were purchased from Sigma–Aldrich (St. Louis, MO, USA).

### 2.5. β-Glucosidase Activity Assay

β-glucosidase activity was measured as described previously [38]. Briefly, cells from the cultures of selected strains were harvested by centrifugation at 17,000 × *g* for 5 min at 4 °C, washed twice with 500 μL of 50 mM phosphate buffer at pH 6.0, and resuspended in 500 μL of the same buffer. The cell suspensions were sonicated using a Vibra-Cell Ultrasonic Liquid Processor (Sonics & Materials Inc., Newtown, CT, USA) to prepare them for the intracellular β-glucosidase activity assay. Then, 90 μL of each sample was mixed with 90 μL of 5 mM p-nitrophenyl-β-D-glucopyranoside and incubated at 37 °C for 20 min. Reactions were stopped by adding 100 μL of ice-cold 0.5 M Na_2_CO_3_ solution, and then centrifuged at 17,000 × *g* for 1 min at 4 °C. For evaluation of β-glucosidase activity, the yellow color of the p-nitrophenyl was determined spectrophotometrically at 405 nm using the SPECTROstar Nano (BMG Labtech, Ortenberg, Germany). The β-glucosidase activity of all samples was expressed as unit/mL, where β-glucosidase converts 1 μmol of p-nitrophenol per minute under assay conditions.

### 2.6. Fermentation of FRG

**Figure 1:**
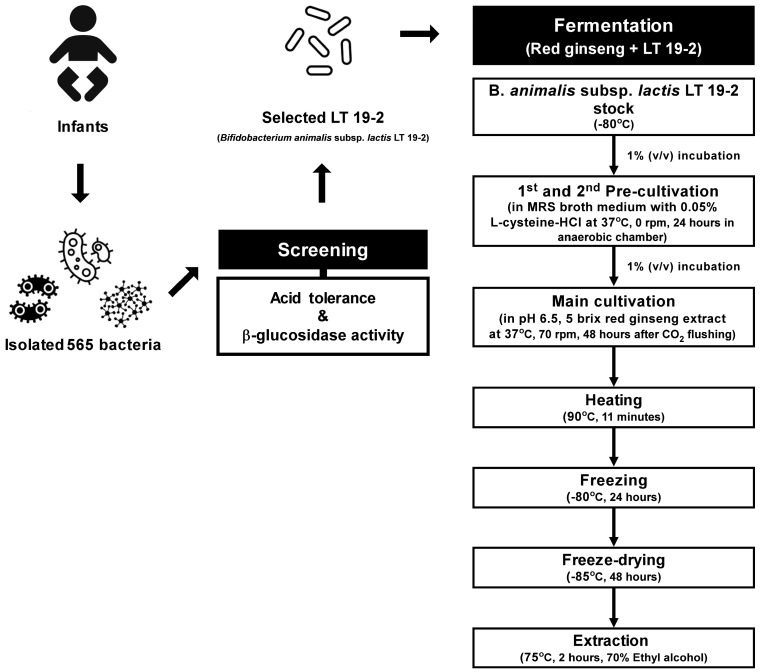
The scheme of fermented red ginseng (FRG) production.

The RG was purchased from Nonghyup (Seoul, Korea) and fermented as described in Figure 1. Briefly, *B. animalis* subsp. *lactis* LT 19-2 was pre-cultivated twice in MRSC at 37 °C for 24 h in anaerobic conditions. The pre-culture was inoculated into fresh RG extract (RGE) medium to a final concentration of 1% (v/v) corresponding to approximately 1 × 10^6^ CFU/mL, and fermented at 37 °C, 70 rpm for 48 h. Before fermentation, the RGE medium was prepared by dissolving Korean RGE (Nonghyup, Seoul, Korea), in distilled water and by adjusting the pH to 6.5 with 1 N NaOH. Samples were collected during fermentation for cell viability and pH measurement. The fermented RG using LT 19-2 (FRG) was heated in a hot water bath at 90 °C for 11 min to kill LT 19-2 in FRG and stored overnight at −80 °C prior to freeze-drying. The freeze-dried sample was extracted with 70% ethyl alcohol at 75 °C for 2 h and kept at −80 °C until functional evaluation of FRG.

### 2.7. Cell Culture and Macrophage Stimulatory Activity

RAW264.7 murine macrophages (Korea Cell Line Bank, Seoul, Korea) were cultured in Dulbecco’s modified Eagle’s medium (DMEM) containing 10% fetal bovine serum (FBS) and 1% penicillin-streptomycin. Cells were incubated at 37 °C under 5% CO_2_. RAW 264.7 macrophages (0.9 × 10^5^ cells) were precultured in 24-well plates for 24 h, and the adherent cells were stimulated with 25 or 50 μg/mL of fermented red ginseng (FRG) for 6 h. The culture media were individually collected and used to quantify the macrophage-induced cytokines, tumor necrosis factor (TNF)-α, and interleukin (IL)-6. The cytokine levels were determined using corresponding enzyme-linked immunosorbent assay (ELISA) kits according to the manufacturer’s protocols (R&D Systems, Minneapolis, MN, USA).

### 2.8. Immunoblot Analysis

RAW264.7 cells (2.25 × 10^6^ cells/mL) were treated with FRG for 0.5 h. Then, cells were rinsed, scraped off the bottoms of wells, and collected in radioimmunoprecipitation assay (RIPA) lysis buffer containing protease and phosphatase inhibitors (Sigma-Aldrich, USA). After centrifugation of the lysate, supernatants were collected and quantified using the Pierce BCA Protein Assay Kit (Thermo Fisher Scientific, Waltham, MA, USA). Proteins were separated by 10% SDS-PAGE and transferred to a nitrocellulose membrane (Bio-Rad, USA). After blocking in 5% skim milk in tris-buffered saline (TBS) containing 0.1% Tween 20 (TBST) for 1 h, the membrane was incubated with the corresponding antibody overnight at 4 °C. After washing with TBST, the membrane was incubated with HRP-conjugated secondary antibody for 1 h, and membranes were visualized using Western Lightning Plus-ECL reagents (PerkinElmer, Waltham, MA, USA).

### 2.9. Splenocyte Proliferation Assay

The experimental protocol was approved by the Animal Care and Use Committee of Seoul National University (SNU 170220-2-2). Spleens were washed with Roswell Park Memorial Institute (RPMI) 1640 medium, crushed to isolate the splenocytes, and passed through a 200-mesh stainless steel sieve to obtain a homogeneous cell suspension. The spleen suspension was washed twice with RPMI 1640-FBS medium and the recovered splenocytes were resuspended in ACK buffer (Gibco, Grand Island, NY, USA) for 3 min to remove erythrocytes. The splenocytes were finally resuspended in 10% FBS RPMI 1640 and cultured in 96-well plates treated with FRG. After 48 h, cultured splenocytes were counted using CellTiter-Glo^®^ Luminescent Cell Viability Assay kits according to the manufacturer’s protocols (Promega, Madison, WI, USA).

### 2.10. Isolation of Bone Marrow-Derived Macrophages

Primary macrophages were isolated from the bone marrow of six-week-old female C57BL/6 mice. The mice were purchased from Young Bio (Seongnam, Korea). Bone marrow cells were isolated from the femurs and tibias. Cells were differentiated for six days in DMEM/F-12 (Corning, New York, NY, USA), containing FBS, 1% penicillin and streptomycin, and 40 ng/mL M-CSF (PeproTech, Rocky Hill, NJ, USA). The medium was replenished every other day. All experimental protocols were approved by the Institutional Animal Care and Use Committee (SNU-170220-2-2) of Seoul National University, Seoul, Korea.

### 2.11. Statistical Analysis

Statistical analyses were performed using SPSS software (SPSS Inc., Chicago, IL, USA). Data were expressed as the mean  ±  standard error of the mean (SEM) and analyzed by a Student’s *t*-test or one-way analysis of variance (ANOVA) followed by Duncan’s Multiple Range Test. A *p*-value of < 0.05 was used to indicate statistical significance.

## 3. Results

### 3.1. Isolation and Screening of 565 Strains for Discovery of Novel Probiotics

**Figure 2:**
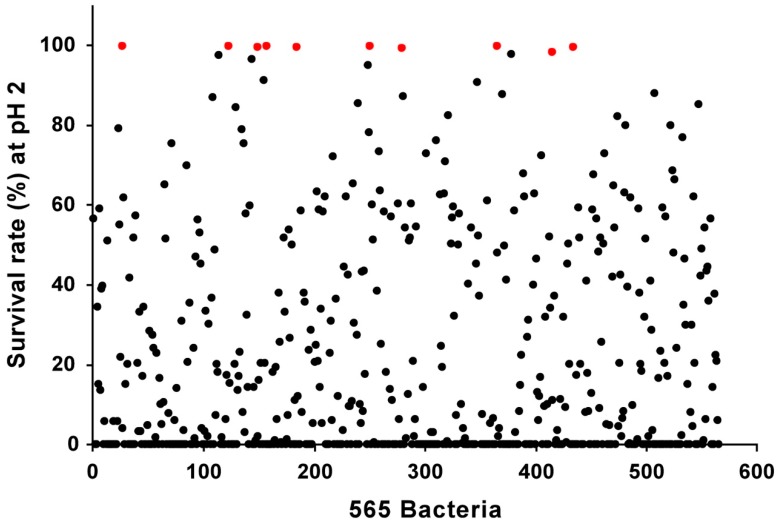
Survival rate of 565 bacteria at pH 2. Red marks show top 10 strains, which have high acid tolerance at pH 2.

**Figure 3:**
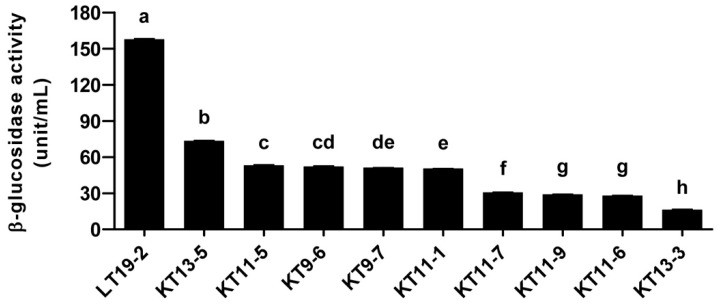
LT 19-2 showed the highest β-glucosidase activity in selected 10 strains. Data are represented as mean ± SEM values of three independent experiments. Mean values with different letters over the bars indicate significant difference (*p* < 0.05).

In order to identify novel bacterial strains that can be used to increase the aglycone content, we screened bacteria isolated from infant feces based on their acid tolerance and β-glucosidase activity (Figure 1). Using selective media, we isolated 565 strains of LAB and Bifidobacteria from infant feces (Supplementary Table S1). Acid tolerance is an important factor to consider when discovering probiotic strains, for surviving the acidic environment of the stomach is crucial for the viability of the bacteria [39]. When developing functional foods based on fermentation, the process also requires acid tolerance for the bacteria being used, as low acid tolerance of the LAB will lead to reduced viability and cessation of the fermentation process [40]. Therefore, for the initial screening, we tested 565 strains for their ability to survive under acidic conditions. We selected the top 10 strains with the strongest acid tolerance, which displayed near 100% viability under pH 2 for 2 h (Figure 2 and Table 1). All of the selected strains were found to be *Bifidobacterium animalis* subsp. *lactis* based on their 16S rRNA analysis (Table 2). As the aglycone derivatives have been known to exert more potent health-beneficial effects compared to their corresponding glycosides [23,24,25], we next focused on the β-glucosidase activity of the isolated strains. We examined the β-glucosidase activity of the selected 10 strains and found that LT 19-2 had the highest β-glucosidase activity among the tested strains (Figure 3). Antibiotic susceptibility was assessed to analyze the safety of LT 19-2. LT 19-2 exhibited high sensitivity to ampicillin, tetracycline, erythromycin, gentamicin, and chloramphenicol (Supplementary Table S2). Collectively, LT 19-2 showed strong acid tolerance among the 565 strains isolated and displayed superior β-glucosidase activity compared to other acid tolerant strains. Thus, we selected LT 19-2 for further evaluation on fermentation-based bioactivity improvement.

### 3.2. Fermented Red Ginseng Using LT 19-2 Shows Enhanced Immunomodulatory Function and Activation of MAPKs and NF-κB Signaling Pathways

**Figure 4:**
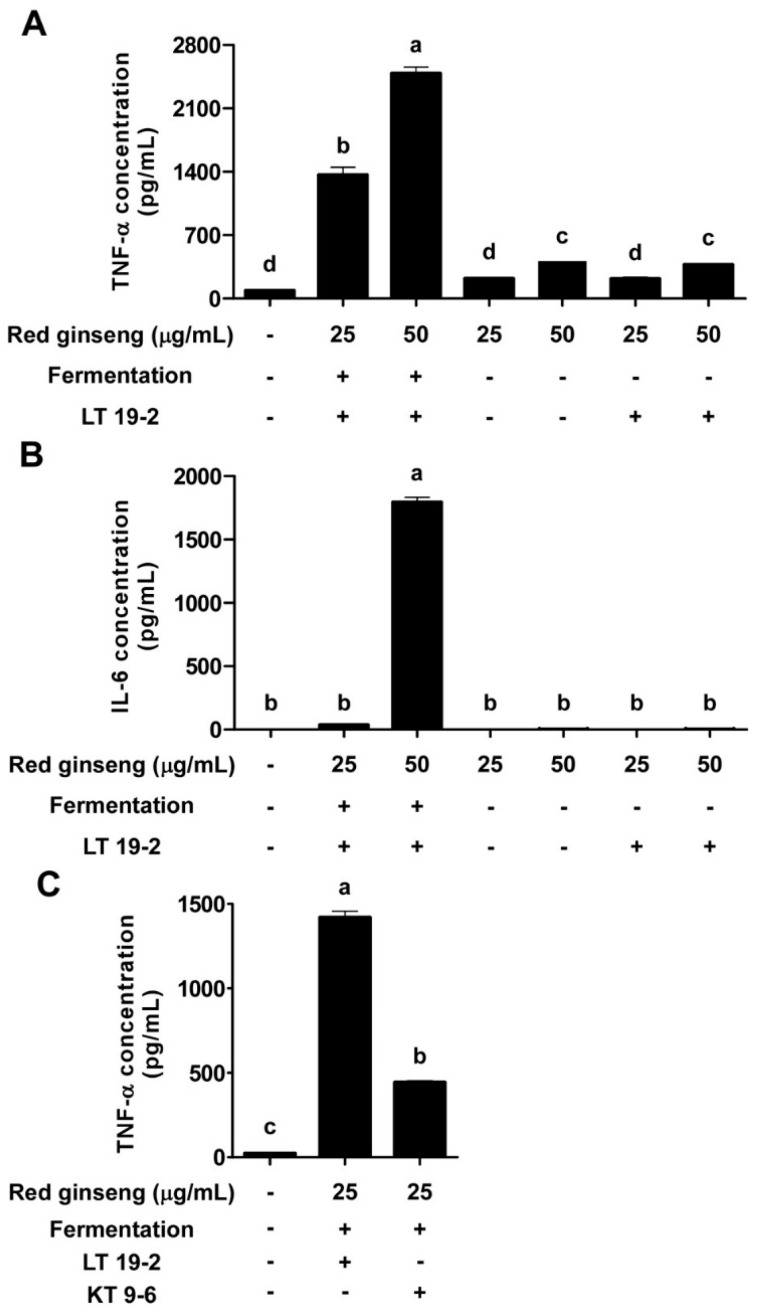
Effects of FRG on TNF-α and IL-6 production. (**A**), (**B**) RAW264.7 cells were treated with FRG at the indicated concentrations, and the media was collected after 6 h for (**A**) TNF-α and (**B**) IL-6 analysis. TNF-α and IL-6 levels were measured as described in the Materials and Methods section. (**C**) RAW264.7 cells were treated with FRG or fermented red ginseng using KT 9-6 at the indicated concentrations, and the media was collected after 6 h. TNF-α levels were measured as described in the Materials and Methods section. Data are represented as mean ± SEM values of three independent experiments. Mean values with different letters over the bars indicate a significant difference (*p* < 0.05).

**Figure 5:**
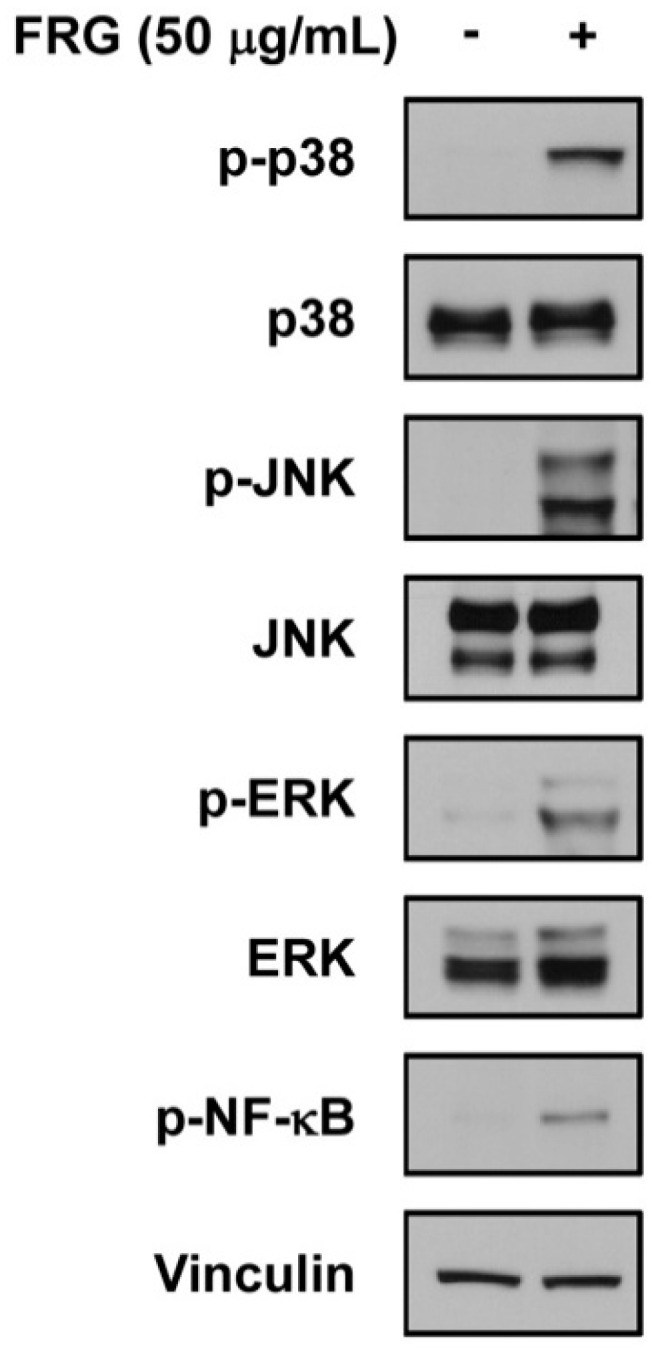
Involvement of p38, ERK, JNK, and NF-κB in the immunomodulatory effect of FRG. RAW264.7 cells were treated with FRG for 0.5 h. Cells were lysed, and proteins were subjected to immunoblotting. Vinculin was used as a loading control.

To investigate whether fermentation using LT 19-2 can alter the aglycone pool and improve its bioactivity, we decided to conduct fermentation using LT 19-2 and red ginseng (Figure 1). Red ginseng has been widely used as a functional food with various health beneficial effects and ginsenosides have been known to be responsible for much of its bioactivity [12,13,41,42]. Previous reports have suggested that removal of sugar moieties from ginsenosides may improve its bioactivity, and thus bacterial strains with β-glucosidase activity have been proposed to increase aglycone content of ginsenosides [27,43,44]. Based on this rationale we fermented red ginseng with LT 19-2, which was chosen for its strong acid tolerance and high β-glucosidase activity (Figure 1). To assess the immunomodulatory function of fermented red ginseng using LT 19-2 (FRG), we analyzed TNF-α and IL-6 secretion levels in RAW264.7. Macrophages play an important role in maintaining homeostasis and protecting from foreign pathogens [45]. During the innate immune response, TNF-α and IL-6 are produced by activated macrophages to control host defense and inflammation [45,46,47]. Treatment with FRG increased TNF-α and IL-6 production (Figure 4A,B). Importantly, the enhancement of immune function by FRG was significantly stronger compared to that of the original red ginseng (RG) or RG without LT 19-2 (Figure 4A,B). To further confirm the effect of fermentation by LT 19-2, we generated fermented RG using a different strain with relatively lower β-glucosidase activity from our screening. KT 9-6 was chosen for its acid tolerance and relatively low β-glucosidase activity (Table 1 and Figure 3). Treatment of RG fermented with LT 19-2 or KT 9-6 both increased TNF-α levels (Figure 4C). However, RG fermented with LT 19-2 (FRG) displayed significantly higher activity compared to RG fermented with KT 9-6 (Figure 4C) suggesting that fermentation using strains with high β-glucosidase activity correlates with higher bioactivity. Evaluation endotoxin levels showed FRG to be free of endotoxin contamination (Supplementary Table S3). To identify the molecular mechanism related to the observed immunomodulatory effect of FRG, we investigated mitogen-activated protein kinases (MAPKs) and NF-κB signaling pathways due to their connection with macrophage activation [48,49]. Treatment with FRG activated p38, c-Jun N-terminal kinase/stress-activated protein kinases (JNK/SAPKs), and extracellular signal–regulated kinases-1/2 (ERK1/2) (Figure 5). In addition, FRG upregulated NF-κB phosphorylation (Figure 5), suggesting that the immunomodulatory function of FRG could involve the activation of MAPKs and NF-κB signaling pathways.

### 3.3. FRG Displays Immune-Stimulatory Effects in Primary Immune Cells

**Figure 6:**
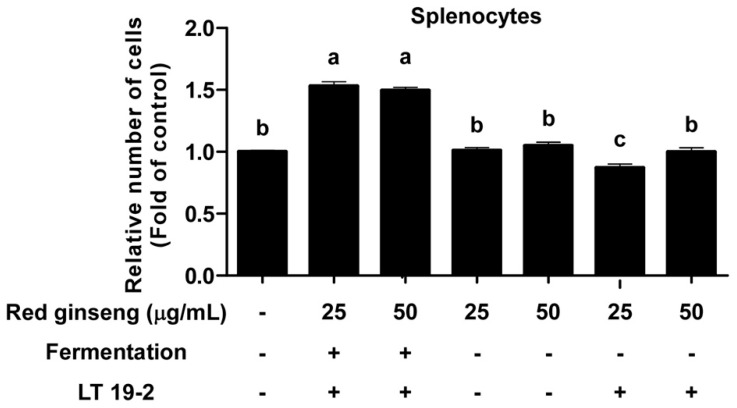
The effect of FRG on splenocyte proliferation. Splenocyte cells were isolated from mice and seeded in a 96-well plate. Celltiter-glo luminescent cell viability assay were performed 48 h after FRG treatment. Data are represented as mean ± SEM values of three independent experiments. Mean values with different letters over the bars indicate a significant difference (*p* < 0.05).

**Figure 7:**
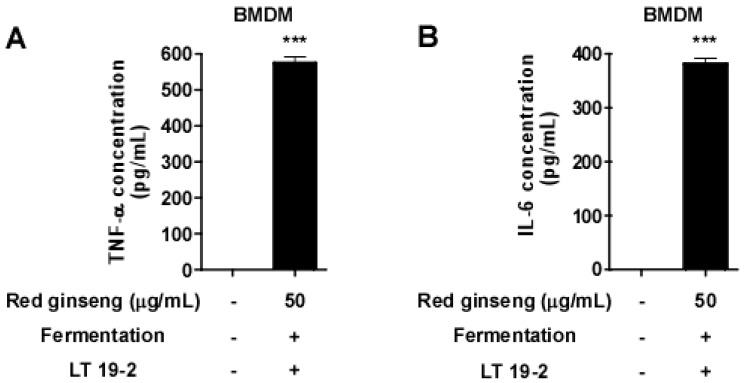
The effect of FRG on TNF-α and IL-6 production in bone marrow-derived macrophages (BMDMs). Primary BMDMs were isolated from mice and differentiated for six days. Media was collected for TNF-α and IL-6 analysis 24 h after FRG treatment. (**A**) TNF-α and (**B**) IL-6 levels were measured as described in the Materials and Methods section. *** *p* < 0.001, significant difference between FGR treated group and control (*n* = 3).

To further confirm the immune-stimulatory potential of FRG, we examined the effect of FRG using primary splenocytes. Treatment of FRG on primary splenocytes promoted splenocyte proliferation (Figure 6). Importantly, the effect of FRG was more potent compared to RG in increasing splenocyte proliferation (Figure 6). Next, we isolated primary bone marrow-derived macrophages (BMDMs) and treated FRG. FRG was capable of inducing TNF-α and IL-6 production even in BMDMs (Figure 7), suggesting that the immune-stimulatory activity of FRG can be also generated in primary immune cells. These results suggest that fermentation with LT 19-2 can improve the immunomodulatory function of red ginseng.

### 3.4. Analysis of the Ginsenoside Content in FRG Showed an Increase in Aglycone Metabolites

**Figure 8:**
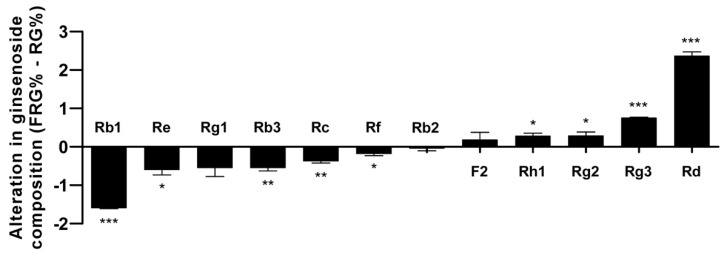
Alteration in relative composition of ginsenosides in red ginseng (RG) and FRG. The relative proportion (%) of ginsenoside contents was analyzed. The differences are shown by subtracting the % value of RG from % value of FRG. Positive values mean an increase in relative content in FRG compared to RG and negative values mean a decrease in relative content in FRG compared to RG. * *p* < 0.05; ** *p* < 0.01; *** *p* < 0.001, significant differences in relative content between FRG and RG (*n* = 3).

The primary purpose of the study was to identify probiotic strains that can increase the aglycone content through β-glucosidase activity. Therefore, we investigated whether fermentation using LT 19-2 led to alteration in the profile of major ginsenosides (Figure 8). Rb1, Rc, and Re are major ginsenosides found in ginseng and red ginseng, and removal of their sugar moieties has been suggested to increase their bioactivity in previous studies [12,13,23,50,51]. Comparison of FRG and RG showed that FRG has a reduction in Rb1, Re, Rc, and Rb3 content while having an increase in Rd, Rh1, F2, and Rg3 levels (Figure 8). Rd is the aglycone of Rb1 and Rc; Rh1 is the aglycone of Re; Rg3 is the aglycone of Rb3: Proving that fermentation using LT 19-2 can remove sugars from ginsenosides leading to an increase in the aglycone pool (Figure 8).

## 4. Discussion

In the current study, the amount of functional aglycones was increased by fermentation with LT 19-2. Fermentation of food products using bacterial strains can lead to novel functional foods with enhanced bioactivity, by enlarging the pool and content of aglycones [12,13,42,50,52]. For example, many studies have shown that the ginsenoside Rg3, the aglycone of ginsenoside Rb1, exhibits a superior anti-cancer effect than that of Rb1 [23,42,52]. Compound K, the aglycone form of the ginsenoside Rb1, has been shown to exert superior effects on colorectal cancer [24], artherogenesis [13], inflammation [50], and liver injury [12]. Thus, we screened 565 strains of LAB and Bifidobacterium with the purpose of discovering novel probiotic strains for fermenting RG (Figure 1). We selected the strain LT 19-2, because of its high acid tolerance and potent β-glucosidase activity. Fermentation of RG, which is a well-known immune-enhancing product, with LT 19-2 increased the immunomodulatory effect compared to RG. To further confirm the effect of fermentation using LT 19-2, we generated two different fermented RG products using either LT 19-2 or KT 9-6. The strain KT 9-6 was used for its relatively lower β-glucosidase activity compared to that of LT 19-2. RG fermented with LT 19-2 (FRG) showed higher immune-stimulatory effects than that of the RG fermented by bacteria having lower β-glucosidase activity (i.e., KT 9-6), suggesting that the enhanced immunomodulatory effect after fermentation has a positive correlation with the β-glucosidase activity of the strain used in fermentation. We suppose that the high β-glucosidase activity might play an important role in enhancing the immunomodulatory effect of red ginsengs as it can effectively increase the aglycone pool. However, as multiple metabolic processes occur during the fermentation process, it is also possible that unknown activities of LT 19-2 could be contributing to the enhanced bioactivity. Thus, it would be interesting to identify other characteristics of the strain, in addition to β-glucosidase activity, that might be critical in improving the functionality during bioconversion of food materials.

We analyzed the changes in some well-known ginsenosides. Interestingly, the content of ginsenoside Rd, Rh1, F2, and Rg3 was increased after fermentation, whereas that of ginsenoside Rb1 Re, Rc, and Rb3 was decreased. In a previous study, ginsenoside Rd was reported to enhance immune responses in a mouse immunization model [14], suggesting that increased levels of ginsenoside Rd could be at least partially responsible for the immune stimulatory effects of FRG. In addition, we measured the content of only 12 major ginsenosides. It is possible that fermentation of RG leads to the removal of sugar residues from other ginsenosides. Therefore, the increased immunomodulatory effect of fermented RG might result from increased levels of ginsenoside Rd, Rh1, F2, and Rg3 as well as increased levels of additional ginsenoside metabolites.

Collectively, our study demonstrates that screening probiotics based on targeted enzyme activity could allow selective biotransformation of bioactive compounds. LT 19-2 should be further studied as a novel probiotic for human consumption. Additionally, using strains such as LT 19-2 for fermentation could lead to the generation of novel functional foods with enhanced aglycone contents and bioactivity.

## Figures and Tables

**Table 1:** Acid tolerance of 10 selected strains.

Strains	Survival Rate (%) at pH 2 ^1^
	2.0
KT13-5	100.00
KT9-7	100.00
KT11-6	100.00
KT11-9	99.97
KT11-1	99.80
KT13-3	99.79
KT9-6	99.74
KT11-5	99.65
LT19-2	99.41
KT11-7	98.35

^1^ Data were obtained after 2 h of incubation.

**Table 2:** Identification of selected strains isolated from infant feces.

Strains	Shape	Color	Morphology	Catalase	Gram	16S rRNA Identification
KT9-7	Round	White	Short rod	-	+	*Bifidobacterium animalis* subsp. *lactis*
KT11-1	Round	White	Short rod	-	+	*Bifidobacterium animalis* subsp. *lactis*
KT9-6	Round	White	Short rod	-	+	*Bifidobacterium animalis* subsp. *lactis*
KT11-5	Round	White	Short rod	-	+	*Bifidobacterium animalis* subsp. *lactis*
KT11-6	Round	White	Short rod	-	+	*Bifidobacterium animalis* subsp. *lactis*
KT11-7	Round	White	Short rod	-	+	*Bifidobacterium animalis* subsp. *lactis*
KT11-9	Round	White	Short rod	-	+	*Bifidobacterium animalis* subsp. *lactis*
KT13-3	Round	White	Short rod	-	+	*Bifidobacterium animalis* subsp. *lactis*
KT13-5	Round	White	Short rod	-	+	*Bifidobacterium animalis* subsp. *lactis*
LT19-2	Round	White	Short rod	-	+	*Bifidobacterium animalis* subsp. *lactis*

## Supplementary Materials

The following are available online at https://www.mdpi.com/2072-6643/11/7/1481/s1, Table S1: Bacteria isolated infant feces, Table S2: Antibiotic susceptibility of Bifidobacterium animalis subsp. lactis LT 19-2, Table S3: Examination of endotoxin levels in FRG.
