# Supplementary material for: Enhancing Immunomodulatory Function of Red Ginseng Through Fermentation Using Bifidobacterium animalis Subsp. lactis LT 19-2
Source: Nutrients. 2019 Jun 28;11(7):1481. doi: 10.3390/nu11071481 (PMC6682942; doi:10.3390/nu11071481)
Supplement: Supplementary file 1 [file nutrients-11-01481-s001.pdf]

Supplementary Table 1. Bacteria isolated infant feces.

Strains	Shape	Color	Morphology	Catalase	Gram	Survival rate (%) at pH[a]
						2.0
LT19-3	Round	White	Short rod	-	+	22.38
KT7-1	Round	White	Short rod	-	+	62.10
KT7-3	Round	White	Short rod	-	+	59.42
KT7-8	Round	White	Short rod	-	+	54.40
KT9-1	Round	White	Long rod	-	+	45.20
KT15-3	Round	White	Long rod	-	+	43.56
KT21-5	Round	White	Short rod	-	+	53.80
KT21-7	Round	White	Short rod	-	+	63.05
KT22-2	Round	White	Short rod	-	+	41.04
KT22-3	Round	White	Short rod	-	+	38.09
KT22-6	Round	White	Short rod	-	+	62.22
HT2-3	Round	White	Rod	-	+	58.40
LT6-2	Round	White	Short rod	-	+	24.66
LT8-11	Round	White	Short rod	-	+	14.28
LT13-5	Round	White	Short rod	-	+	41.86
LT14-6	Round	White	Short rod	-	+	37.95
LT15-4	Round	White	Short rod	-	+	39.99
LT16-3	Round	White	Short rod	-	+	73.02
LT18-1	Round	White	Long rod	-	+	50.99
KT1-1	Round	White	Short rod	-	+	65.28
KT8-7	Round	White	Short rod	-	+	67.92
KT8-8	Round	White	Short rod	-	+	78.18
KT8-11	Round	White	Short rod	-	+	57.83
KT12-3	Round	White	Short rod	-	+	42.12

KT13-6	Round	White	Short rod	-	+	73.31
KT13-7	Round	White	Short rod	-	+	82.21
KT14-1	Round	White	Short rod	-	+	75.42
KT16-6	Round	White	Long rod	-	+	61.08
KT17-3	Round	Yellow	Short rod	-	+	63.65
KT17-4	Round	Yellow	Short rod	-	+	90.65
KT18-2	Round	White	Thick rod	-	+	82.36
KT19-1	Round	White	Short rod	-	+	87.20
KT22-7	Round	White	Short rod	-	+	56.78
HT3-2	Round	White	Rod	-	+	61.80
HT3-3	Round	White	Rod	-	+	52.26
HT3-4	Round	White	Rod	-	+	62.02
HT4-5	Round	White	Rod	-	+	50.96
HT5-1	Round	White	Rod	-	+	54.48
HT5-4	Round	White	Rod	-	+	58.62
HT5-5	Round	White	Rod	-	+	73.01
HT6-1	Round	White	Rod	-	+	50.10
HT7-5	Round	White	Rod	-	+	44.48
HT9-5	Round	White	Rod	-	+	49.88
HT11-1	Round	White	Short rod	-	+	41.11
HT20-1	Round	White	Rod	-	+	85.30
HT20-2	Round	White	Rod	-	+	51.52
LM3-2	Irregular	Yellow	Rod	-	+	20.78
LM9-5	Round	White	Rod	-	+	32.03
LM10-7	Round	White	Rod	-	+	54.21
LM12-6	Round	White	Short rod	-	+	59.05
LM19-1	Round	White	Rod	-	+	6.42

LM19-2	Round	Yellow	Rod	-	+	33.39
LM19-4	Round	Yellow	Rod	-	+	57.84
LM21-5	Round	Yellow	Rod	-	+	34.29
LM21-6	Round	Yellow	Rod	-	+	30.01
LM25-4	Round	White	Rod	-	+	9.56
LM25-5	Irregular	White	Rod	-	+	27.42
LT6-3	Round	White	Short rod	-	+	34.60
LT6-4	Round	White	Short rod	-	+	5.25
LT6-6	Round	White	Short rod	-	+	4.72
LT6-7	Round	White	Short rod	-	+	32.33
LT7-2	Round	White	Short rod	-	+	20.54
LT8-7	Round	White	Short rod	-	+	16.24
LT8-8	Round	White	Short rod	-	+	66.50
LT8-9	Round	White	Short rod	-	+	17.11
LT9-5	Round	White	Short rod	-	+	20.33
LT20-3	Round	White	Bifid rod	-	+	1.47
LT20-4	Round	White	Short rod	-	+	3.55
KT1-3	Round	White	Short rod	-	+	47.95
KT2-2	Round	White	Medium rod	-	+	64.97
KT2-3	Round	White	Medium rod	-	+	58.26
KT3-6	Round	White	Short rod	-	+	70.93
KT15-5	Round	White	Short rod	-	+	52.97
KT15-6	Round	White	Long rod	-	+	51.89
KT15-7	Round	White	Short rod	-	+	33.34
KT16-3	Round	White	Short rod	-	+	62.97
KT16-7	Round	White	Short rod	-	+	45.22
KT16-8	Round	White	Short rod	-	+	59.03

KT17-2	Round	Yellow	Short rod	-	+	72.22
KT19-4	Round	White	Short rod	-	+	46.42
KT22-4	Round	White	Short rod	-	+	62.84
KT22-5	Round	White	Short rod	-	+	51.90
HT4-4	Round	White	Rod	-	+	61.76
HT5-3	Round	White	Rod	-	+	57.15
HT6-2	Round	White	Rod	-	+	45.24
HT7-3	Round	White	Rod	-	+	54.98
HT7-4	Round	White	Rod	-	+	50.35
HT9-1	Round	White	Rod	-	+	76.99
HT9-4	Round	White	Rod	-	+	51.44
HT9-6	Round	White	Rod	-	+	57.18
HT10-5	Round	White	Rod	-	+	48.23
HT10-6	Round	White	Rod	-	+	51.69
HT10-9	Round	White	Rod	-	+	54.22
HT10-10	Round	White	Rod	-	+	58.76
HT19-5	Round	White	Rod	-	+	62.20
HT23-1	Irregular	White	Long rod	-	+	80.05
HT23-3	Irregular	White	Long rod	-	+	72.44
HT25-4	Round	White	Rod	-	+	0
LM5-3	Irregular	Yellow	Rod	-	+	8.12
LM6-4	Irregular	Yellow	Rod	-	+	27.54
LM6-9	Irregular	Yellow	Rod	-	+	35.96
LM7-1	Round	White	Rod	-	+	31.04
LM7-2	Round	White	Rod	-	+	28.75
LM7-7	Round	White	Rod	-	+	47.14
LM8-6	Round	White	Rod	-	+	85.44

LM10-4	Round	White	Short rod	-	+	0
LM10-5	Round	White	Short rod	-	+	87.72
LM10-6	Round	White	Rod	-	+	76.30
LM13-1	Round	White	Short rod	-	+	60.30
LM15-3	Round	Yellow	Rod	-	+	57.24
LM15-4	Round	White	Rod	-	+	56.68
LM15-6	Round	Yellow	Rod	-	+	84.57
LM15-7	Round	White	Rod	-	+	68.56
LM18-4	Round	White	Rod	-	+	60.28
LM18-8	Round	Yellow	Rod	-	+	30.30
LM19-5	Round	White	Rod	-	+	17.26
LM19-6	Round	White	Rod	-	+	4.86
LM19-7	Round	Yellow	Rod	-	+	39.61
LM19-8	Round	White	Rod	-	+	69.80
LM20-5	Round	Yellow	Rod	-	+	35.54
LM22-2	Round	White	Rod	-	+	41.29
LM22-3	Round	White	Rod	-	+	48.02
LM25-1	Irregular	White	Rod	-	+	42.60
LM25-2	Round	White	Rod	-	+	20.22
LM25-3	Round	White	Rod	-	+	17.36
LM26-1	Round	White	Rod	-	+	38.96
LM26-2	Irregular	White	Rod	-	+	49.98
LT1-3	Round	White	Short rod	-	+	20.21
LT1-4	Round	White	Short rod	-	+	23.36
LT3-4	Round	White	Short rod	-	+	28.36
LT3-5	Round	White	Short rod	-	+	56.65
LT4-1	Round	White	Short rod	-	+	33.89

LT4-2	Round	White	Short rod	-	+	75.44
LT4-5	Round	White	Short rod	-	+	25.23
LT6-1	Round	White	Short rod	-	+	20.95
LT7-1	Round	White	Short rod	-	+	67.70
LT7-5	Round	White	Short rod	-	+	14.28
LT7-6	Round	White	Short rod	-	+	5.24
LT7-8	Round	White	Short rod	-	+	18.30
LT14-1	Round	White	Short rod	-	+	44.64
LT14-2	Round	White	Short rod	-	+	50.40
LT19-1	Round	White	Short rod	-	+	87.10
LT19-2	Round	White	Short rod	-	+	99.41
LT19-4	Round	White	Short rod	-	+	17.18
LT20-1	Round	White	Bifid rod	-	+	17.95
LT20-2	Round	White	Short rod	-	+	62.58
KT1-2	Round	White	Short rod	-	+	59.39
KT13-4	Round	White	Short rod	-	+	0
KT16-4	Round	White	Short rod	-	+	58.88
KT16-5	Round	White	Short rod	-	+	51.70
KT20-3	Round	White	Short rod	-	+	56.40
KT20-4	Round	White	Short rod	-	+	51.92
KT20-5	Round	White	Short rod	-	+	46.43
KT21-9	Round	White	Short rod	-	+	51.80
HT9-7	Round	White	Rod	-	+	42.32
HT20-3	Round	White	Rod	-	+	79.31
HT23-2	Irregular	White	Long rod	-	+	43.16
HT23-4	Irregular	White	Long rod	-	+	56.59
HT26-3	Round	White	Rod	-	+	37.87

LM9-4	Round	White	Short rod	-	+	50.38
LM9-6	Round	White	Rod	-	+	65.07
LM13-2	Round	White	Short rod	-	+	51.98
LM19-3	Round	Yellow	Rod	-	+	20.33
LM20-1	Round	White	Rod	-	+	0
LM20-2	Round	Yellow	Rod	-	+	0
LM22-5	Round	White	Rod	-	+	79.06
LT15-5	Round	White	Short rod	-	+	21.86
LT22-1	Round	White	Bifid rod	-	+	26.72
KT7-2	Round	White	Short rod	-	+	60.16
KT9-6	Round	White	Short rod	-	+	99.74
KT9-7	Round	White	Short rod	-	+	100.72
KT11-1	Round	White	Short rod	-	+	99.80
KT11-2	Round	White	Short rod	-	+	96.57
KT11-3	Round	White	Short rod	-	+	79.93
KT11-4	Round	White	Short rod	-	+	97.68
KT11-5	Round	White	Short rod	-	+	99.65
KT11-6	Round	White	Short rod	-	+	100.69
KT11-7	Round	White	Short rod	-	+	98.35
KT11-8	Round	White	Short rod	-	+	97.45
KT11-9	Round	White	Short rod	-	+	99.97
KT13-1	Round	White	Short rod	-	+	0
KT13-3	Round	White	Short rod	-	+	99.79
KT13-5	Round	White	Short rod	-	+	101.26
KT15-8	Round	White	Long rod	-	+	54.44
KT20-2	Round	White	Short rod	-	+	58.65
HT10-2	Round	White	Rod	-	+	91.25

HT10-7	Round	White	Rod	-	+	94.99
HT15-2	Round	White	Rod	-	+	63.44
HT15-3	Round	White	Rod	-	+	59.70
HT17-4	Round	White	Rod	-	+	0
HT18-5	Round	White	Rod	-	+	0
HT19-8	Round	White	Rod	-	+	32.04
LM4-1	Irregular	Yellow	Rod	-	+	31.88
LM9-3	Round	White	Short rod	-	+	10.63
LM22-1	Round	White	Rod	-	+	88.09
LT1-1	Round	White	Short rod	-	+	0
LT1-2	Round	White	Short rod	-	+	15.25
LT1-5	Round	White	Short rod	-	+	0
LT1-6	Round	White	Short rod	-	+	0
LT2-1	Round	White	Short rod	-	+	0
LT2-2	Round	White	Short rod	-	+	0
LT2-3	Round	White	Short rod	-	+	0
LT3-1	Round	White	Short rod	-	+	7.52
LT3-2	Round	White	Short rod	-	+	10.96
LT3-3	Round	White	Short rod	-	+	15.24
LT4-3	Round	White	Short rod	-	+	0.75
LT4-4	Round	White	Short rod	-	+	3.18
LT5-1	Round	White	Short rod	-	+	15.40
LT5-2	Round	White	Short rod	-	+	1.37
LT5-3	Round	White	Short rod	-	+	0
LT5-4	Round	White	Short rod	-	+	0
LT5-5	Round	White	Short rod	-	+	0
LT6-5	Round	White	Short rod	-	+	5.94

LT7-3	Round	White	Short rod	-	+	20.69
LT7-4	Round	White	Short rod	-	+	3.41
LT7-7	Round	White	Short rod	-	+	0
LT7-9	Round	White	Long rod	-	+	11.26
LT8-1	Round	White	Short rod	-	+	11.19
LT8-2	Round	White	Short rod	-	+	0
LT8-3	Round	White	Short rod	-	+	0
LT8-4	Round	White	Short rod	-	+	0
LT8-5	Round	White	Short rod	-	+	0
LT8-6	Round	White	Short rod	-	+	0
LT8-10	Round	White	Short rod	-	+	0
LT9-1	Round	White	Short rod	-	+	5.24
LT9-2	Round	White	Short rod	-	+	4.70
LT9-3	Round	White	Short rod	-	+	7.44
LT9-4	Round	White	Short rod	-	+	23.04
LT9-6	Round	White	Short rod	-	+	13.61
LT10-1	Round	White	Short rod	-	+	5.12
LT10-2	Round	White	Short rod	-	+	6.65
LT10-3	Round	White	Short rod	-	+	6.34
LT10-4	Round	White	Short rod	-	+	20.54
LT11-1	Round	White	Short rod	-	+	0
LT11-2	Round	White	Short rod	-	+	0.06
LT12-1	Round	White	Short rod	-	+	0.02
LT12-2	Round	White	Short rod	-	+	0.03
LT12-3	Round	White	Short rod	-	+	0.06
LT12-4	Round	White	Short rod	-	+	3.03
LT13-1	Round	White	Short rod	-	+	0

LT13-2	Round	White	Short rod	-	+	0
LT13-3	Round	White	Short rod	-	+	0
LT13-4	Round	White	Short rod	-	+	0
LT14-3	Round	White	Short rod	-	+	0
LT14-4	Round	White	Short rod	-	+	0
LT14-5	Round	White	Short rod	-	+	0
LT15-1	Round	White	Short rod	-	+	0
LT15-2	Round	White	Short rod	-	+	0
LT15-3	Round	White	Short rod	-	+	0
LT15-6	Round	White	Short rod	-	+	0
LT16-1	Round	White	Short rod	-	+	0
LT16-2	Round	White	Short rod	-	+	0.98
LT17-1	Round	White	Long rod	-	+	38.04
LT17-2	Round	White	Short rod	-	+	0
LT17-3	Round	White	Short rod	-	+	0
LT18-2	Round	White	Short rod	-	+	13.70
LT18-3	Round	White	Short rod	-	+	6.57
LT18-4	Round	White	Short rod	-	+	0
LT21-1	Round	White	Bifid rod	-	+	0
LT21-2	Round	White	Bifid rod	-	+	0
KT1-4	Round	White	Short rod	-	+	20.98
KT1-5	Round	White	Short rod	-	+	1.55
KT1-6	Round	White	Short rod	-	+	0.34
KT1-7	Round	White	Long rod	-	+	0
KT1-8	Round	White	Long rod	-	+	0
KT2-1	Round	White	Medium rod	-	+	0
KT2-4	Round	White	Medium rod	-	+	0

KT2-5	Round	White	Medium rod	-	+	0
KT3-1	Round	White	Long rod	-	+	0
KT3-2	Round	White	Short rod	-	+	0
KT3-3	Round	White	Short rod	-	+	0
KT3-4	Round	White	Short rod	-	+	0
KT3-5	Round	White	Short rod	-	+	0
KT7-4	Round	White	Short rod	-	+	0
KT7-5	Round	White	Short rod	-	+	0
KT7-6	Round	White	Short rod	-	+	0
KT7-7	Round	White	Short rod	-	+	0
KT8-1	Round	White	Short rod	-	+	0
KT8-2	Round	White	Long rod	-	+	0
KT8-3	Round	White	Short rod	-	+	0
KT8-4	Round	White	Long rod	-	+	0
KT8-5	Round	White	Short rod	-	+	0
KT8-6	Round	White	Short rod	-	+	0
KT8-9	Round	White	Short rod	-	+	0
KT8-10	Round	White	Short rod	-	+	0
KT8-12	Round	White	Short rod	-	+	0
KT9-2	Round	White	Short rod	-	+	0
KT9-3	Round	White	Long rod	-	+	0
KT9-5	Round	White	Short rod	-	+	0
KT9-8	Round	White	Short rod	-	+	3.06
KT9-9	Round	White	Short rod	-	+	NG
KT9-10	Round	White	Short rod	-	+	16.77
KT9-11	Round	White	Short rod	-	+	0
KT9-12	Round	White	Short rod	-	+	0

KT9-13	Round	White	Short rod	-	+	0
KT9-14	Round	White	Short rod	-	+	38.40
KT10-1	Round	White	Long rod	-	+	0
KT10-2	Round	White	Short rod	-	+	37.15
KT10-3	Round	White	Short rod	-	+	0
KT10-4	Round	White	Long rod	-	+	0
KT10-5	Round	White	Long rod	-	+	0
KT10-6	Round	White	Long rod	-	+	34.48
KT10-7	Round	White	Long rod	-	+	1.79
KT10-8	Round	White	Long rod	-	+	8.04
KT10-9	Round	White	Long rod	-	+	12.97
KT12-1	Round	White	Short rod	-	+	2.40
KT12-2	Round	White	Short rod	-	+	22.34
KT12-4	Round	White	Short rod	-	+	14.37
KT12-5	Round	White	Short rod	-	+	18.20
KT12-6	Round	White	Short rod	-	+	35.63
KT12-7	Round	White	Short rod	-	+	16.59
KT12-8	Round	White	Short rod	-	+	2.16
KT12-9	Round	White	Short rod	-	+	0
KT12-10	Round	White	Short rod	-	+	0
KT13-2	Round	White	Short rod	-	+	0
KT14-2	Round	White	Short rod	-	+	0
KT14-3	Round	White	Short rod	-	+	0
KT14-4	Round	White	Short rod	-	+	0
KT14-5	Round	White	Short rod	-	+	0
KT14-6	Round	White	Short rod	-	+	0
KT14-7	Round	White	Short rod	-	+	0

KT15-1	Round	White	Long rod	-	+	0
KT15-2	Round	White	Short rod	-	+	8.47
KT15-4	Round	White	Short rod	-	+	6.42
KT15-9	Round	White	Long rod	-	+	0
KT15-10	Round	White	Short rod	-	+	0
KT15-11	Round	White	Short rod	-	+	0
KT15-12	Round	White	Short rod	-	+	0
KT16-1	Round	White	Long rod	-	+	0
KT16-2	Round	White	Long rod	-	+	0
KT16-9	Round	White	Short rod	-	+	0
KT16-10	Round	White	Short rod	-	+	0
KT16-11	Round	White	Short rod	-	+	0
KT16-12	Round	White	Short rod	-	+	1.68
KT17-1	Round	Yellow	Short rod	-	+	9.04
KT17-5	Round	Yellow	Short rod	-	+	0
KT17-6	Round	Yellow	Short rod	-	+	0
KT17-7	Round	Yellow	Short rod	-	+	5.80
KT18-1	Round	White	Thick rod	-	+	0
KT19-2	Round	White	Large rod	-	+	4.02
KT19-3	Round	White	Short rod	-	+	0
KT19-5	Round	White	Short rod	-	+	0
KT19-6	Round	White	Large rod	-	+	0
KT19-7	Round	White	Short rod	-	+	3.58
KT19-8	Round	White	Short rod	-	+	0.55
KT19-9	Round	White	Short rod	-	+	15.20
KT20-1	Round	White	Short rod	-	+	0
KT20-6	Round	White	Short rod	-	+	0

KT20-7	Round	White	Short rod	-	+	0
KT20-8	Round	White	Short rod	-	+	0.66
KT21-2	Round	White	Short rod	-	+	0
KT21-4	Round	White	Short rod	-	+	0
KT21-6	Round	White	Short rod	-	+	0
KT21-8	Round	White	Short rod	-	+	0
KT22-1	Round	White	Short rod	-	+	0
KT22-8	Round	White	Short rod	-	+	0
HT1-1	Round	White	Rod	-	+	0
HT1-2	Round	White	Rod	-	+	0
HT1-3	Round	White	Rod	-	+	0
HT1-4	Round	White	Rod	-	+	0
HT1-5	Round	White	Rod	-	+	0
HT1-6	Round	White	Rod	-	+	0
HT2-1	Round	White	Rod	-	+	4.05
HT2-2	Round	White	Rod	-	+	2.11
HT2-4	Round	White	Rod	-	+	6.02
HT2-5	Round	White	Rod	-	+	14.86
HT2-6	Round	White	Rod	-	+	0
HT3-1	Round	White	Rod	-	+	0
HT3-5	Round	White	Rod	-	+	0
HT4-1	Round	White	Rod	-	+	0
HT4-2	Round	White	Rod	-	+	0
HT4-3	Round	White	Rod	-	+	0
HT5-2	Round	White	Rod	-	+	0
HT6-3	Round	White	Rod	-	+	0
HT7-1	Round	White	Rod	-	+	0

HT7-2	Round	White	Rod	-	+	0
HT7-6	Round	White	Rod	-	+	0
HT7-7	Round	White	Rod	-	+	0
HT7-8	Round	White	Rod	-	+	0
HT7-9	Round	White	Rod	-	+	0
HT7-10	Round	White	Rod	-	+	0
HT8-1	Round	White	Rod	-	+	0
HT8-2	Round	White	Rod	-	+	0
HT8-3	Round	White	Rod	-	+	0
HT9-2	Round	White	Rod	-	+	0
HT9-3	Round	White	Rod	-	+	0
HT10-8	Round	White	Rod	-	+	11.23
HT11-2	Round	White	Short rod	-	+	30.49
HT12-1	Round	White	Short rod	-	+	2.01
HT12-2	Round	White	Short rod	-	+	6.23
HT12-3	Round	White	Short rod	-	+	0
HT12-4	Round	White	Short rod	-	+	0
HT12-5	Round	White	Short rod	-	+	0
HT12-6	Round	White	Short rod	-	+	0
HT13-1	Round	White	Short rod	-	+	0
HT14-1	Round	White	Short rod	-	+	0
HT14-2	Round	White	Short rod	-	+	0
HT14-3	Round	White	Short rod	-	+	0
HT14-4	Round	White	Short rod	-	+	0
HT14-5	Round	White	Short rod	-	+	0
HT14-6	Round	White	Short rod	-	+	0
HT14-7	Round	White	Short rod	-	+	0.56

HT14-8	Round	White	Short rod	-	+	0
HT14-9	Round	White	Short rod	-	+	0
HT15-1	Round	White	Rod	-	+	0
HT15-4	Round	White	Rod	-	+	0
HT15-5	Round	White	Rod	-	+	0
HT16-1	Round	White	Rod	-	+	0
HT16-2	Round	White	Rod	-	+	0
HT16-3	Round	White	Rod	-	+	0
HT16-4	Round	White	Rod	-	+	13.97
HT16-5	Round	White	Rod	-	+	0.25
HT16-6	Round	White	Rod	-	+	8.36
HT16-7	Round	White	Rod	-	+	0
HT17-1	Round	White	Rod	-	+	0
HT17-2	Round	White	Rod	-	+	14.47
HT17-3	Round	White	Rod	-	+	17.57
HT17-5	Round	White	Rod	-	+	0
HT17-6	Round	White	Rod	-	+	0
HT18-1	Round	White	Rod	-	+	0
HT18-2	Round	White	Rod	-	+	0
HT18-3	Round	White	Rod	-	+	0
HT18-4	Round	White	Rod	-	+	0
HT18-6	Round	White	Rod	-	+	20.50
HT18-7	Round	Yellow	Rod	-	+	39.66
HT19-1	Round	White	Rod	-	+	59.78
HT19-2	Round	White	Rod	-	+	40.25
HT19-3	Round	White	Rod	-	+	27.04
HT19-4	Round	White	Rod	-	+	24.11

HT19-6	Round	White	Rod	-	+	12.15
HT19-7	Round	White	Rod	-	+	30.04
HT20-4	Round	White	Rod	-	+	0
HT20-6	Round	White	Rod	-	+	0
HT20-7	Round	White	Rod	-	+	0
HT21-1	Round	White	Rod	-	+	0
HT21-2	Round	Yellow	Rod	-	+	0
HT21-3	Round	Yellow	Rod	-	+	0
HT21-4	Round	Yellow	Rod	-	+	0
HT21-5	Round	Yellow	Rod	-	+	0
HT21-6	Round	White	Rod	-	+	24.08
HT22-1	Round	Yellow	Rod	-	+	10.24
HT22-2	Round	Yellow	Rod	-	+	18.10
HT22-3	Round	White	Rod	-	+	5.17
HT23-5	Irregular	White	Long rod	-	+	42.56
HT24-1	Round	White	Rod	-	+	36.65
HT24-2	Round	White	Rod	-	+	48.75
HT24-3	Round	White	Rod	-	+	0
HT24-4	Round	White	Rod	-	+	0
HT25-1	Round	White	Rod	-	+	0
HT25-2	Round	White	Rod	-	+	0
HT25-3	Round	White	Rod	-	+	0
HT25-5	Round	White	Rod	-	+	0
HT26-1	Round	White	Rod	-	+	0
HT26-2	Round	White	Rod	-	+	33.14
HT26-4	Round	White	Rod	-	+	0
LM1-1	Irregular	Yellow	Rod	-	+	0

LM1-2	Irregular	Yellow	Rod	-	+	0
LM2-1	Irregular	Yellow	Rod	-	+	0
LM3-3	Irregular	Yellow	Rod	-	+	7.39
LM4-2	Irregular	Yellow	Rod	-	+	0.95
LM5-1	Irregular	Yellow	Rod	-	+	6.41
LM5-2	Irregular	Yellow	Rod	-	+	14.33
LM6-1	Irregular	Yellow	Rod	-	+	0
LM6-3	Irregular	Yellow	Rod	-	+	1.12
LM6-5	Irregular	Yellow	Rod	-	+	0
LM6-6	Irregular	Yellow	Rod	-	+	0
LM6-7	Irregular	Yellow	Rod	-	+	0
LM6-8	Irregular	Yellow	Rod	-	+	0
LM6-10	Irregular	Yellow	Rod	-	+	24.10
LM7-3	Round	White	Rod	-	+	2.16
LM7-4	Round	White	Rod	-	+	6.04
LM7-5	Round	White	Rod	-	+	25.05
LM7-6	Round	White	Rod	-	+	5.74
LM7-8	Round	White	Rod	-	+	1.86
LM8-1	Round	White	Rod	-	+	23.30
LM8-2	Round	White	Rod	-	+	19.32
LM8-3	Irregular	White	Rod	-	+	0
LM8-4	Round	White	Rod	-	+	31.25
LM8-5	Irregular	White	Rod	-	+	13.12
LM8-7	Irregular	White	Rod	-	+	37.32
LM8-8	Round	White	Short rod	-	+	31.02
LM8-9	Round	White	Rod	-	+	49.10
LM8-10	Round	White	Short rod	-	+	36.42

LM9-1	Round	White	Short rod	-	+	43.64
LM9-2	Round	White	Rod	-	+	4.01
LM9-7	Round	White	Short rod	-	+	12.12
LM9-8	Round	White	Short rod	-	+	20.14
LM9-9	Irregular	White	Short rod	-	+	1.02
LM9-10	Round	White	Rod	-	+	28.78
LM10-1	Round	White	Rod	-	+	0
LM10-2	Round	White	Rod	-	+	0
LM10-3	Round	White	Rod	-	+	0
LM10-8	Round	White	Short rod	-	+	5.38
LM10-9	Round	White	Rod	-	+	3.50
LM11-1	Round	White	Short rod	-	+	7.44
LM11-2	Round	White	Short rod	-	+	12.54
LM11-3	Round	White	Short rod	-	+	9.78
LM11-4	Round	White	Short rod	-	+	20.06
LM11-5	Round	White	Short rod	-	+	0
LM12-1	Round	White	Short rod	-	+	6.02
LM12-2	Round	White	Short rod	-	+	3.68
LM12-3	Round	White	Short rod	-	+	23.76
LM12-4	Round	White	Short rod	-	+	1.22
LM12-5	Round	White	Short rod	-	+	4.65
LM12-7	Round	White	Short rod	-	+	6.12
LM12-8	Round	White	Short rod	-	+	32.38
LM13-3	Round	White	Short rod	-	+	3.06
LM13-4	Round	White	Short rod	-	+	8.33
LM13-5	Round	White	Short rod	-	+	8.04
LM13-6	Round	White	Short rod	-	+	20.88

LM14-1	Round	White	Short rod	-	+	2.17
LM14-2	Round	White	Short rod	-	+	9.61
LM14-3	Round	White	Short rod	-	+	20.18
LM14-4	Round	White	Short rod	-	+	4.02
LM14-5	Round	White	Short rod	-	+	10.22
LM14-6	Round	White	Short rod	-	+	12.19
LM15-1	Round	White	Rod	-	+	8.01
LM15-2	Round	White	Rod	-	+	18.08
LM15-5	Round	White	Rod	-	+	0
LM16-1	Round	White	Rod	-	+	0
LM16-2	Round	White	Rod	-	+	0
LM16-3	Round	White	Rod	-	+	0
LM16-4	Round	White	Rod	-	+	0
LM16-5	Round	White	Rod	-	+	0
LM16-6	Round	White	Rod	-	+	10.13
LM16-7	Round	White	Rod	-	+	2.14
LM17-1	Round	White	Rod	-	+	3.22
LM17-2	Round	Yellow	Rod	-	+	17.28
LM17-3	Round	Yellow	Rod	-	+	0.16
LM17-4	Round	Yellow	Rod	-	+	16.94
LM17-5	Round	Yellow	Rod	-	+	0
LM17-6	Round	Yellow	Rod	-	+	0
LM17-7	Round	White	Rod	-	+	0
LM17-8	Round	White	Rod	-	+	0
LM18-1	Round	White	Rod	-	+	0
LM18-2	Round	Yellow	Rod	-	+	0
LM18-3	Round	Yellow	Rod	-	+	0

LM18-5	Round	Yellow	Rod	-	+	0
LM18-6	Round	Yellow	Rod	-	+	0
LM18-7	Round	Yellow	Rod	-	+	0
LM20-3	Round	White	Rod	-	+	34.96
LM20-4	Round	Yellow	Rod	-	+	20.06
LM21-1	Round	White	Rod	-	+	7.87
LM21-2	Round	White	Rod	-	+	3.25
LM21-3	Round	White	Rod	-	+	9.32
LM21-4	Round	White	Rod	-	+	11.09
LM21-7	Round	White	Rod	-	+	25.77
LM22-4	Round	White	Rod	-	+	19.30
LM23-1	Round	White	Rod	-	+	25.66
LM23-2	Round	More white	Rod	-	+	0
LM23-3	Round	More white	Rod	-	+	23.02
LM23-4	Round	White	Rod	-	+	14.04
LM23-5	Round	White	Rod	-	+	9.56
LM24-1	Round	White	Rod	-	+	0
LM24-2	Round	White	Rod	-	+	0
LM24-3	Round	White	Rod	-	+	0
LM24-4	Round	White	Rod	-	+	0
LM24-5	Round	White	Rod	-	+	8.32
LM26-3	Irregular	White	Rod	-	+	0
LM26-4	Irregular	White	Rod	-	+	0
LM26-5	Round	White	Rod	-	+	0
LM26-6	Round	White	Rod	-	+	0
KT21-1	Round	White	Short rod	-	+	0

LM3-1	Irregular	Yellow	Rod	-	+	10.09
LM6-2	Irregular	Yellow	Rod	-	+	20.32

Supplementary Table 2. Antibiotic susceptibility of *Bifidobacterium animalis* subsp. *lactis* LT 19-2.

Strain	MIC [1] (mg/L) of antibiotics					
	Amp	Kan	Tet	Ery	Gen	Chl
LT19-2	≤ 1	> 128	4	≤ 1	64	≤ 1

[1] MIC, Minimal inhibitory concentration; Amp, ampicillin; Kan, kanamycin; Tet, tetracycline; Ery, erythromycin; Gen, gentamicin; Chl: chloramphenicol.

Supplementary Table 3. Examination of endotoxin levels in FRG.

Standard Endotoxin conc. (EU/mL)	Absorbance (405nm)
100	0.149
10	0.130
1	0.125
0.1	0.093
0.01	0.070
0	0.066

Sample	Endotoxin conc. (EU/mL)
FRG	N.D[1]

[1]N.D, Not detected
